# EU-HTA—Why Do Words Matter?

**DOI:** 10.3390/jmahp14020031

**Published:** 2026-05-18

**Authors:** Mondher Toumi, Bruno Falissard, Steven Simoens, Maarten Postma, Marta Wilk, Laurent Boyer, Renato Bernardini, Stefano Capri, Jaime Espin, Jürgen Wasem, Pascal Auquier

**Affiliations:** 1 CEReSS/UR3279—Health Services Research and Quality of Life Center, Aix-Marseille University, 13385 Marseille, France; laurent.boyer@univ-amu.fr (L.B.); pascal.auquier@univ-amu.fr (P.A.); 2 CESP, INSERM U1018, Université Paris-Saclay, 94800 Villejuif, France; bruno.falissard@gmail.com; 3 Department of Pharmaceutical and Pharmacological Sciences, Katholieke Universiteit Leuven, 3000 Leuven, Belgium; steven.simoens@kuleuven.be; 4 Department of Health Sciences, University Medical Center Groningen, University of Groningen, 9700 AB Groningen, The Netherlands; m.j.postma@rug.nl; 5 Department of Economics, Econometrics & Finance, Faculty of Economics & Business, University of Groningen, 9713 AB Groningen, The Netherlands; 6 Center of Excellence in Higher Education for Pharmaceutical Care Innovation, Universitas Padjadjaran, Bandung 40132, Indonesia; 7 Division of Pharmacology & Therapy, Faculty of Medicine, Universitas Airlangga, Surabaya 60131, Indonesia; 8 Clever-Access, 30-415 Krakow, Poland; marta.wilk@clever-access.com.com; 9 Department of Biomedical and Biotechnological Sciences (BIOMETEC), Section of Pharmacology, University of Catania, 95124 Catania, Italy; bernardi@unict.it; 10 School of Economics and Management, Cattaneo-LIUC University, 21053 Castellanza, Italy; scapri@liuc.it; 11 Andalusian School of Public Health/Escuela Andaluza de Salud Pública (EASP), 18011 Granada, Spain; jaime.net2010@gmail.com; 12 CIBER of Epidemiology and Public Health (CIBERESP), 28029 Madrid, Spain; 13 Instituto de Investigación Biosanitaria, 18012 Granada, Spain; 14 Institute of Healthcare Management, Universität Duisburg-Essen, 45127 Essen, Germany; juergen.wasem@medman.uni-due.de

## 1. Introduction

Words matter because they have profound power to shape thoughts, emotions, actions, and social realities. They influence our thoughts and actions daily by shaping our perceptions and behaviors at both conscious and subconscious levels. Beyond words, semantics are concerned with how language conveys meaning and how people understand and use that meaning in each particular context.

The words and semantics used in the European Health Technology Assessment (EU-HTA) Regulation [1], guidance documents, guidelines, and implementation acts play a crucial role in shaping our understanding, interpretation, and conceptualization of EU-HTA reality. Our perception of the EU-HTA Regulation and its implementation will be significantly influenced by the terminology employed by the European Commission (EC) and the Member State Health Technology Assessment Coordination Group (MS-HTA-CG). Several terms and expressions used in official documents, such as the EU-HTA Regulation and guidance documents or guidelines, appear semantically imprecise or misleading, as they may create systematic bias and inaccurate vision of the EU-HTA. They may distort general understanding of Health Technology Assessment (HTA) as well.

This paper analyzes words and semantics used by the EC and MS-HTA-CG in publicly available materials that appear misused. This editorial does not report such misuses in a systematic way, but underlines several of the most prominent ones to raise awareness on this important phenomenon.

## 2. A Misleading Scope and Process Naming

Extensive use of the abbreviation EU-HTA aims to suggest there is an EU-centralized HTA process, even though it is a Member State (MS) collaboration supported by an EU secretariate and restricted to limited aspects of the HTA concept.

- The EU Regulation 2021/2282 [1] is commonly referred to as the “EU-HTA Regulation”, implying a single, EU-driven centralized HTA process. In reality, the regulation only establishes cooperation of the MS on certain aspects (notably Joint Scientific Consultation (JSC) and Joint Clinical Assessment (JCA)) of the HTA, but ignores critical parts of HTA such as health economics assessment and the appraisal process. The scope of the law is narrow, decontextualized, and judgment-free, focusing on clinical evidence assessment instead of encompassing a broad, centralized HTA framework.
- As stated in the regulation [1], the purpose of the EU-HTA Regulation is to facilitate EU MS cooperation. EC as a facilitator provides a secretariate platform to secure the flow of information between stakeholders and secure the proper administrative process according to the EU state-of-the-art administrative process. Therefore, a more adequate term for the EU-HTA Regulation would be EU Member State Health Technology Assessment (MS-HTA) cooperation regulation.
- The EU-HTA Regulation terminology could also mislead individuals into expecting a centrally managed appraisal, a definitive HTA decision for all countries, when, in fact, the EU level provides only a joint clinical assessment (without value judgments or contextualization). This can create false expectations and confusion about roles and responsibilities. As one analysis noted, the term “EU-HTA” has been widely adopted and “perceived as representing an EU-HTA process, which does not exist”.
- The name “EU-HTA Regulation” goes well beyond the collaboration scope that primarily covers two topics: the JSC related to clinical evidence development, and the JCA concerning the evaluation of clinical evidence. The EU-HTA focuses on clinical evidence and its assessment against a set of reference documents. This is known in epistemology as the assessment of the ontic component of the experimental clinical evidence. Due to its limited scope in contextualization and impartial approach, the EU-HTA addresses only a small segment of the overall clinical evidence assessment. A more accurate designation would be “EU HTA Joint Clinical Ontic Component Assessment.” Integrating previous comments would advocate for naming the regulation the EU MS Ontic Clinical HTA Cooperation Regulation. As it assesses deviation versus guidance documents and guidelines, the word audit shall be used instead of assessment. The current terminology creates confusion between the specific clinical focus of the regulation and the broader concept of HTA. This misconception has become deeply ingrained.

## 3. Is the “Degree of Certainty of Relative Effectiveness” What the JCA Subgroup Assesses?

This regulation is designed to evaluate the degree of certainty of relative effectiveness. Further clarification is necessary to define the terms “effectiveness”, “certainty”, degree”, and “relative”, including their specific meanings and implications in the context of the EU-HTA Regulation.

- Effectiveness refers to efficacy measured in routine clinical practice, in contrast to efficacy usually measured under experimental conditions (clinical trials). Despite the guideline advising against a hierarchy of evidence, the Health Technology Assessment Coordination Group (HTA-CG) has placed randomized controlled trials (RCTs) at the top of the evidence hierarchy while positioning real-world evidence at the bottom [2]. To address this apparent contradiction, the HTA-CG states in its guidance on outcomes that, “For simplicity, effectiveness is the term used to describe efficacy or effectiveness throughout this document” [3]. In practice, the JCA will primarily conduct relative efficacy assessments using experimental clinical trials, with exceptional instances of effectiveness assessment. The primary mission of the HTA is to assess clinical effectiveness, distinct from clinical efficacy, which falls within the scope of regulatory agencies. The authors of the guidance will be assessing relative efficacy based on RCTs in most cases, so the guidance document may have to be reviewed.
- The certainty level is the output of this exercise. In experimental medical and biological sciences, scientific reasoning typically operates under conditions of uncertainty, with certainty being rarely achieved or targeted. This asymmetry indicates that uncertainty is the standard epistemic condition, while certainty is the exception. Researchers primarily assess the uncertainty associated with their findings or the research they review. In epistemology, certainty and uncertainty are not symmetrical measures; they have an asymmetrical relationship grounded in the functions of knowledge and doubt. Therefore, translating certainty from uncertainty is neither scientifically nor epistemologically sound and defensible in empirical sciences. Policy decision-makers aspire to make decisions based on certainty. They seek an optimal certain situation to justify their decisions through available robust knowledge. This translation from uncertainty to certainty is also observed in the EU for food safety. According to EU food safety training, scientists are trained to assess uncertainty and should then report the certainty level [4]. Similarly, EU-HTA assessors evaluate uncertainty and manage it through various sensitivity analysis models. They then present their conclusions at a certainty level as required by the EU-HTA Regulation. This creates the impression that we operate in an environment where certainty is possible, while in reality, it remains highly uncertain.
- The degree of certainty indicates the level of confidence attributed to empirical findings, integrating statistical evidence, study design, methodological integrity, and practical significance to assess the reliability and validity of research conclusions. By definition, the degree of certainty involves a nuanced judgment reflecting a subjective evaluation of the collective evidence and its appraisal. This does not comply with the EU-HTA Regulation [1], which requires JCA to be impartial, decontextualized, judgment-free, and to report the certainty of relative effectiveness. Additionally, while a degree indicates the level of certainty, there is no clear guidance on how to estimate it transparently and reproducibly. It is also unclear whether this degree will be reported as a continuous variable (e.g., 0 to 1), an ordinal scale, or a categorical interval. How the large amount of information will be translated into a degree remains unknown, yet this is essential for the outcome of the JCA. There are no rules that allow for integrating multiple pieces of information into a single score qualifying the degree (of certainty). So far, the “raison d’être” of the JCA operates as a black box that has not been clarified. It will likely be reported as a narrative with risk of divergent interpretation at the MS level.
- The term “relative” in relative effectiveness pertains to the comparison of benefits and harms between two interventions. This terminology has been consistently used in EU documents and is not new to this regulation. To the authors’ knowledge, it appears officially in EU documents in EU high-level pharmaceutical forum outcome reporting. Conversely, in the United States, the term “comparative effectiveness” is preferred as it more clearly indicates a comparison. The term “relative” can be ambiguous, potentially referring either to the ratio of an efficacy outcome between two medicinal products (preferred by the Institute for Quality and Efficiency in Healthcare (IQWIG)) or to comparison of an efficacy outcome between two medicinal products (preferred by the National Authority for Health (HAS)). The dual meaning of “relative” may lead to confusion regarding whether to report the comparative gain or the rate ratio of gain. This casts doubt on being able to compare relative gain. While both absolute and relative differences are acceptable, the choice between them is not neutral. An absolute difference of 10% in response rate is 10% whatever the comparator response is. But in relative effect, if the response rate is 20% for the comparator, 10% absolute gain will be 50% in relative value, but if the comparator response rate is 50%, the 10% absolute gain is 20% in relative value. In relative terms, the gain is dependent on comparator performance. So using relative or an absolute difference of 10% in response rate is always 10%, regardless of the comparator’s response. However, in relative terms, if the comparator’s response rate is 20%, a 10% absolute gain equals a 50% relative gain. If the comparator’s response rate is 50%, a 10% absolute gain equals a 20% relative gain. Hence, relative gains depend on the comparator’s performance, affecting how the gain is represented. Using comparative wording is clearer and more accurate. Absolute gain or relative gain measure will have an impact on the cognitive representation of the gain. The wording comparative would be more appropriate and straightforward, avoiding the confusion associated with relative dual meaning. There could be a risk that “relative” could lead assessors and Health Technology Developers (HTDs) to focus on the ratio rather than absolute difference, while “comparative” may avoid this misunderstanding.

## 4. Evidence-Based Medicine (EBM) and State-of-the-Art—Methodological Fundamentalism Disguised as Scientific Rigor

Along the course of all these documents and more specifically the guidance and guidelines, the wording aims to shape the belief of an extremely high level of scientific methodology, referring to gold standard, adequacy, and true effect (as opposed to the wrong one, violation of assumptions, meaningful benefit, unbiased and robust evidence claims), all of which suggest that the MS-EU-HTA CG is pursuing the highest quality and unique indisputable methodological truth.

- The terms EBM and state-of-the-art science are mentioned very frequently in the EU-HTA Regulation (7 times in total) [1], yet the guidelines and guidance documents were not developed using EBM principles, which could also apply in developing these guidelines. Essential EBM steps—such as framing the question and searching, finding, and assessing evidence—were not followed. Despite repeated references to EBM, its principles were not applied, potentially leading readers to accept unsupported claims through repetition alone. “Repeating a message often enough makes it become a truth” is a well-established propaganda process.
- The gold standard as the only option. The most pervasive issue across EU HTA documents is the presentation of methodological approaches as scientifically mandated rather than contextually appropriate. The documents consistently use “gold standard” to describe RCT, stating that “RCTs provided they are well designed and have low risk of bias, are the gold standard for informing estimates of treatment effectiveness”. This terminology ignores that these choices are based on an underlying epistemological preference for a positivist approach rather than a constructivist one. For example, the National Institute of Health and Care Excellence (NICE) in their guidance emphasizes “appropriate” evidence rather than “gold standard” evidence, acknowledging that the most suitable methodology depends on the clinical question and available alternatives [5,6]. The “gold standard” framing becomes particularly problematic when applied to complex interventions, rare diseases, Advanced Therapy Medicinal Products (ATMPs), or innovative therapeutic approaches where RCTs may be impossible or ethically questionable. By establishing this hierarchy based on the concept of gold standard, the EU guidance justifies the exclusion of valuable evidence sources that are the only option in some circumstances. However, this is justified by MS-HTA-CG, which interprets the EU-HTA Regulation as decontextualized and judgment-free in a broad sense.
- Concerning binary thinking about evidence quality, EU HTA documents repeatedly employ “adequate” versus “inadequate” dichotomies in describing evidence quality, creating oversimplified binary classifications that do not reflect the spectrum of evidence validity. Terms like “adequate RCT data,” “adequate comparisons,” and “adequate analysis” appear throughout the guidance without clear criteria for what constitutes “adequacy.” This contrasts with the International Network of Agencies for Health Technology Assessment (INAHTA) in light of evidence medicine that recognizes that evidence quality exists on a continuum. The binary approach becomes particularly problematic because it gives assessors inappropriate discretionary power to classify evidence as “adequate” or “inadequate” without any transparent criteria and irrespective of context. While the ultimate goal of the assessor is to assess the degree of certainty of relative effectiveness, nowhere in the regulation does it specify how inadequate translates in the degree of certainty. This wording creates the opportunity for arbitrary decision supported by inadequacy wording. This could lead to systematic bias against innovative study designs or therapeutic areas where traditional RCT methodology is challenging to implement.
- Subjective qualifiers presented as objective criteria. The EU guidance frequently uses subjective qualifiers disguised as objective methodological requirements. The term “meaningful” appears repeatedly without clear definition—studies are dismissed as “unlikely to provide a meaningful estimate of treatment effectiveness” without specifying what constitutes meaningfulness. The concept of meaningfulness is well established and could be seen either as the Minimum Clinical Important Difference (MCID) and is addressed either mathematically or through an expert consensus estimating what is the efficacy gain need to consider if it is clinically meaningful. Or it could be considered as the output of the deliberative process that will determine the clinical meaningfulness of the efficacy gain. International HTA agencies handle this more transparently. NICE explicitly defines “clinically meaningful differences” through stakeholder engagement and expert consultation processes. Canada’s Drug Agency/L’Agence des medicaments du Canada (CDA-AMC) requires applicants to justify their proposed minimal clinically important differences. The Dental and Pharmaceutical Benefits Agency (TLV) in Sweden uses structured approaches to define disease severity and treatment benefit thresholds. These agencies recognize that determinations of clinical meaningfulness are inherently subjective and require transparent deliberative processes. The EU guidance’s use of undefined “meaningful” criteria creates a veneer of objectivity over what are essentially value judgments, potentially masking important policy decisions as technical assessments. This touches the cornerstone of the EU-HTA Regulation that is considered as decontextualized and judgment-free and, at the same time, assesses meaningfulness that requires judgment and context to be understandable.
- False claims about evidence objectivity. Perhaps most concerning is the repeated use of “unbiased” and “robust” evidence claims throughout the documents. The guidance promises “unbiased estimation of the relative treatment effectiveness” and identifies “the most robust evidence” as coming from study designs [7]. This language implies achievable states of complete objectivity that are methodologically naive. Empirical evidence generation navigates between practical feasibility and scientific rigor. When designing an experiment, researchers attempt to minimize biases and the impact of these biases on critical outcomes. So, empirical research cannot be free of bias. The academic HTA literature consistently emphasizes epistemic humility—the recognition that all evidence is limited and potentially biased. The *International Journal of Technology Assessment in Health Care* regularly publishes papers on managing uncertainty and bias in HTA, emphasizing the importance of transparent acknowledgment of limitations. Unbiased and robust evidence claim in HTA is a chimera. But because JCA is expected to be decontextualized and judgment-free and aims to assess the ontic component, it needs to promise unbiased and robust evidence to ensure the credibility of its deliverables.
- Categorical exclusions. Similarly, the EU guidance makes numerous categorical exclusions using “inappropriate” and “unreliable” classifications without acknowledging contextual factors or methodological evolution. Methods are labeled as “inappropriate to…” or “not appropriate” without considering specific therapeutic contexts or alternative approaches.

## 5. Scientific Misconception Legitimated Through Words and Peremptory Definitions

- The guidance document on statistical analysis and multiplicity implicitly endorses the hypothetico-deductive framework approach, although this is not explicitly stated [8].
- Nonetheless, it also notes that for certain MSs, post hoc analyses may be considered more significant than ad hoc analyses.
- Post hoc analyses are generally viewed as hypothesis-generating rather than hypothesis-testing. The nominal *p* value refers to the outcome reported from a statistical test (such as a *t*-test or chi-square test) without adjustments for multiple comparisons or considerations regarding the underlying assumptions of the test validity. If the assumptions of a significance test are not met, the nominal *p* value should not be considered actionable or meaningful from a statistical perspective.
- In conclusion, in this guidance document, a nominal *p* value de facto refers to a statistically invalid *p* value. Referring to such a *p* value as “nominal” without clearly indicating to which framework it pertains, and whether underlying statistical test assumptions are violated or not, may create the false impression that the *p* value is valid when, in fact, it is not.
- Internal validity is described in the guidance on clinical trial validity as the absence of biases, and a tool has been suggested to standardize the assessment of internal validity [2]. However, internal validity encompasses more than just the risk of biases. It involves establishing causality between the intervention being tested and the observed outcomes in an experiment, which includes factors such as population homogeneity, for example. Defining internal validity solely in terms of bias risk may overlook its broader methodological aspects and could lead to the perception that internal validity is a straightforward concept measurable by a standardized tool. This confers a false belief of robustness.
- In the same guidance [2], external validity is portrayed as simple to assess, typically described narratively and using the Population Intervention Comparison Outcome (PICO) framework. However, this overlooks the extensive literature highlighting the complexity of evaluating external validity and the many tools developed for this purpose, potentially leading to the mistaken impression that assessment is standardized and straightforward. This also confers a false belief of robustness.

## 6. Guidelines and Guidance Searching for the Difference

The HTA-CG produced six guidance documents and two guidelines to help assessors in reviewing evidence submitted by HTD and preparing the JCA report. The availability of guidance and guidelines went unnoticed by the experts in the field interviewed at the European Access Academy conference in Berlin (the authors’ personal experience). Additionally, the difference between guidance and guidelines is not defined in EU-HTA documentation. Guidelines are formal, evidence-based, and systematically developed recommendations designed to standardize and optimize clinical care or policies using the best available evidence. Guidance refers to less formal, often expert opinion-based advice or recommendations that provide direction in situations where evidence may be limited, emerging, or rapidly changing. This distinction is important for policymakers to understand the reliability and intended use of these documents. However, a review of the JCA guidance documents and guidelines does not reveal clear distinctions in how they were developed or their intended use. The topic for guidelines concerns indirect treatment comparisons, which has varying acceptance in HTA; some HTA bodies, such as those in France and Germany, do not favor indirect treatment comparisons, while others consider them valuable as additional evidence, particularly in cost-effectiveness analysis. It is not specified whether guidance documents are merely informative while guidelines are more prescriptive, or if both serve similar functions and purposes.

## 7. Conclusions

Even though our analysis is not systematic, this paper has illustrated that terms and concepts identified throughout EU HTA documents serve to create the impression that:

- The EU-HTA Regulation is a centrally managed and operationalized EU process;
- The process is guided by principles of EBM;
- Assessors will adhere to clear, transparent rules, ensuring the reliability and robustness of the JCA report.

In reality, the situation is more nuanced, and such a simplified perspective may foster a false sense of reassurance, potentially deterring thorough scrutiny of the evidence and silencing criticism. Should this occur, the JCA report may not be as actionable at the MS level as anticipated.

Indeed, we argue that these terminological and semantic issues have significant implications for HTA development across the EU, particularly for countries with limited HTA experience. The prescriptive language and false certainty claims could create several problems.

- While the assessment of the degree of certainty is stated as the key outcome of the JCA, it remains a black box concept relying on words framing the illusion that the assessors will provide a quantifiable, robust, and reliable assessment of the degree of certainty of relative effectiveness. Behind the words, there is no element that supports the idea that this assessment will be reliable.
- Methodological fundamentalism disguised as scientific rigor creates an illusion of state-of-the-art practices.
- Exclusion of valuable evidence, through binary classifications and categorical exclusions, could lead to systematic rejection of evidence that established HTA agencies would consider valuable when properly interpreted.
- The certainty language and false precision could encourage overconfident decision-making without adequate acknowledgment of uncertainty.
- The distinction between guidance and guidelines introduces uncertainty regarding their intended application and flexibility, which requires further clarification.

The EU-HTA Regulation should be revised and renamed to clearly reflect its purpose and use precise language rather than vague terms meant to reassure. Guidance documents and guidelines should use accurate wording and remain factual as needed. Using precise vocabulary and reliable claims builds credible documentation for future HTA in Europe. It is essential to exercise care in word choice to ensure that communication effectively conveys accurate and pertinent information.

## Data Availability

No new data is generated.

## Abbreviations

ATMPs	Advanced Therapy Medicinal Products
CDA-AMC	Canada’s Drug Agency/L’Agence des medicaments du Canada
CG	Coordination Group
EBM	Evidence Based Medicine
EC	European Commission
EU-HTA	European Health Technology Assessment
HAS	National Authority for Health
HTA	Health Technology Assessment
HTA-CG	Health Technology Assessment Coordination Group
HTDs	Health Technology Developers
INAHTA	International Network of Agencies for Health Technology Assessment
IQWIG	Institute for Quality and Efficiency in Healthcare
JCA	Joint Clinical Assessment
JSC	Joint Scientific Consultation
NICE	National Institute of Health and Care Excellence
MCID	Minimum Clinical Important Difference
MS	Member State
MS-HTA	Member State Health Technology Assessment
MS-HTA-CG	Member State Health Technology Assessment Coordination Group
PICO	Population Intervention Comparison Outcome
RCT	Randomized Controlled Trial
TLV	Dental and Pharmaceutical Benefits Agency
